# Adherence to infection prevention practices and associated factors among healthcare workers in Northeastern Ethiopia, following the Northern Ethiopia conflict

**DOI:** 10.3389/fpubh.2024.1433115

**Published:** 2024-10-14

**Authors:** Gete Berihun, Adinew Gizeyiatu, Leykun Berhanu, Birhanu Sewunet, Birhanie Ambaw, Zebader Walle, Masresha Abebe, Kassahun Ayele Gasheya

**Affiliations:** ^1^Department of Environmental Health, College of Medicine and Health Sciences, Wollo University, Dessie, Ethiopia; ^2^Department of Public Health, College of Health Sciences, Debre Tabor University, Debre Tabor, Ethiopia; ^3^Department of Occupational Health and Safety, College of Medicine and Health Science, Wollo University, Dessie, Ethiopia

**Keywords:** healthcare workers, infection prevention practices, healthcare facilities, conflict, Northern Ethiopia

## Abstract

**Background:**

In resource-limited areas, especially in conflict-affected settings, managing the risk of hospital-acquired infections is difficult due to the destruction of essential facilities in healthcare settings. The study aimed to assess adherence to Infection prevention practices and associated factors among healthcare workers in Northeastern Ethiopia following the Northern Ethiopia conflict.

**Methods and materials:**

A facility-based cross-sectional study was done with 408 healthcare workers. The survey data was collected using a structured questionnaire based on published articles. Data entry and analysis was done using Epi-Data version 4.6 and SPSS version 25.0, respectively. Binary logistic regression was used to determine the association between dependent and outcome variables, with a cut-off *p* value 0.05 at 95% confidence interval with a *p*-value less than 0.05 and a 95% confidence interval for determining factors associated with adherence to infection prevention practices among study participants.

**Results:**

The study included 408 healthcare workers with a response rate of 96.7%. The majority of participants were women 206 (50.5%), married 250 (61.3%), Orthodox followers 211 (51.7%), and educational status of master holder and above with a master’s degree or higher 177 (43.4%). Slightly more than half, 53.7% (219) of the respondents demonstrated safe infection prevention practices. Respondents who received training on infection prevention practices (AOR = 2.662, 95% CI: 1.361, 5.120) had an active infection prevention committee (AOR = 2.203, 95% CI: 1.359, 3.572), use infection prevention guidelines in working departments (AOR = 2.090, 95% CI: 1.013, 4.312), and access to adequate personal protective equipment (AOR = 2.773, 95% CI: 1.560, 4.929) were factors significantly associated with adherence to infection prevention practices.

**Conclusion:**

Overall, only half of the respondents practiced safe infection prevention practices. Receive training on infection prevention guidelines, presence of active infection prevention committee and working guidelines, and the availability of personal protective equipment were factors of infection prevention. Hence, essential facilities like Personal protective equipment, working guidelines should be supplied by donors.

## Introduction

The conflict in Northern Ethiopia began on November 3, 2020, between the Tigray People Liberation Front (TPLF) and the Federal government of Ethiopia. Since then, it has extended to the nearby Amhara and Afar regions (1, 2). Armed conflict has profound public health implications, leading to both direct and indirect consequences. Direct effects include immediate injuries and deaths caused by utilization of firearms and explosives during conflict (1) and may cause for the death of an estimated 133,750 people every year, excluding indirectly attributable mortality (3). Moreover the indirect effects may cause for decline of the public health through the collapsing of the overall health systems, shortage of medical supplies, displacement of healthcare workers, and disruption of food and clean water supplies. Hence, preventive and curative services of the healthcare facilities become collapsed (1, 3).

Experiences from countries like Syria, South Sudan, Angola, and Nepal showed that healthcare facilities became the targets of armed fighters. For instance, in Syria, 185 health facilities were damaged either knowingly or unknowingly (4). Armed conflicts damage health systems, resulting in a resurgence of preventable infectious diseases. Globally, over three-quarters of the deaths are non-communicable, especially in low and middle-income countries (LMIC), where armed conflict exacerbate long term health issues (5). In LMIC, healthcare facilities face various challenges, including infrastructure, unbalancing of high patient load and low staffs, may complicate the efforts of infection prevention and control measures (6). Armed conflicts further damage the public health infrastructure and hindering healthcare delivery due to destruction of health facilities, shortage of ambulances, medical supplies and equipment’s. (7). Areas affected by conflict usually operate with insufficient staffs which limits the capacity of IPC (8). Hence, implementation of effective IPC programs could potentially reduce the burden of IPC by up to one-third (9).

HAIs are the global public health problem, particularly occur worldwide, particularly prevalent in LMIC. Approximately 10% of hospitalized patients in developed countries and 25% in developing countries experiences HAIs leading to a negative health consequences, including an increased hospital stay, economic burden, morbidity, and mortality (9–11). In Africa, HAIs may affect 3 and 15% in hospitalized patients (12). Adherences to IPC may be hindered by a high workload, insufficient time, and in balanced patient-to-health practitioner ratio. Therefore, implementing various strategies of IPC improvement intervention tactics has been proven to lower HAIs and increase HCWs adherence (13). A systematic review and meta-analysis in Ethiopia revealed that the prevalence of HAI was 16.96% (14). The use of effective and low-cost IPC measures reduces the burden of HCAIs by at least one-third. Despite these strategies are implemented, adherence with standard IPC practices remains very low, especially in LMIC, in which Ethiopia is not exceptional due to the existences of continuous conflict in the region (15).

The Ethiopian Federal Ministry of Health has launched various initiatives to safeguard patients and healthcare workers by establishing standards and guidelines. However, in resource-limited areas, especially in conflict-affected settings, controlling the risk of HCAIs is challenging due to the destruction of essential facilities in healthcare settings. The lack of knowledge and motivation to implement standard infection prevention practices contribute to this challenge (16). In the Northern Ethiopia conflict, there have been numerous reports of destruction and atrocities against health systems and civilians despite, most of the reports have relied on press reports of eyewitness accounts, journalists, humanitarian agencies and official announcements of combatants (1). To the best of our knowledge, there is scanty information on IPC following conflict-affected areas in Ethiopia. Hence, this study aimed to assess adherence with infection prevention practices and associated factors among HCWs in Northeastern Ethiopia, following the Northern Ethiopia conflict.

## Methods and materials

### Study setting and period

The study was carried out in Northeastern Ethiopia. The study catchment has 22 districts. Based on the 2014 population projection report, the total population of the study area was 2,925,559, of which 1,448,174 were males and 1,477,385 were females (17). In terms of healthcare facilities, there were 14 public hospitals, 3 private hospitals, 135 health centers, 496 clinics, and 175 pharmacies (18). The study was conducted from June 1 to June 30, 2022.

### Study design

A facility-based cross-sectional study was carried out using a structured standardized questionnaire based on published articles to assess the adherence to infection prevention practices among healthcare professionals in government healthcare facilities in the South Wollo zone of Northeastern Ethiopia, following the Northern Ethiopia conflict.

### Population (source and study populations)

The source population of the study consisted of all healthcare workers (such as general practitioners, public health officers, nurses, midwives, laboratory technicians and technologists, dentists, anesthetists, ophthalmologists, and cataract surgeons) who were working in governmental healthcare facilities in the South Wollo Zone, Northeastern Ethiopia. On the other hand, the study includes healthcare workers working in the selected governmental healthcare facilities during the study period.

#### Inclusion criteria

The study included healthcare workers with direct contact with at least one of four potential sources, namely patients, medical equipment, linens, and high-risk waste.

#### Exclusion criteria

Healthcare workers with less than six months of working experience, on annual leave, maternity leave, or experiencing illness during the study period were excluded from participating in the study. Additionally, administrative staff and cleaners were also excluded from the study.

### Sample size determination and sampling procedures

The sample size for this study was determined using a single population proportion formula with the following assumptions. Half (50%) of the HCWs were assumed to have good infection prevention practices due to the lack of previous studies following conflicts, a margin of error of 5% and, a 95% confidence interval (CI) were taken into account during the calculation.


n=Z^2∗p∗1−p/d^2

Where: n represents the required sample size, Z is the z-score corresponding to the desired level of confidence (for a 95% confidence level, Z would be 1.96), p is the estimated proportion of the population (50% of the HCWs had good infection prevention practices), **d** is the desired margin of error (the maximum difference allowed between the sample estimate and the true population proportion).

Applying these values, the calculated sample size is 384. Finally, considering for the potential non-response rate of 10%, the final sample size is adjusted to 422.

### Sampling procedures

Regarding the sampling procedures, the initial step involved the selection of 22 governmental healthcare facilities including, health centers, and hospitals by a simple random technique using a lottery method. The number of healthcare providers in each selected facility was obtained from their respective human resource offices. Then, proportional allocation was carried out based on the number of healthcare providers present during the data collection period. Finally, participants were selected using a simple random sampling technique utilizing a sampling frame list provided by their respective human resources offices.

### Study variables

The outcome variable of the study was IPCs which were measured either good or poor. The assessment of healthcare provides infection prevention practices encompassed various measures, including hand hygiene practices, waste management and handling practices, use of PPEs, and vaccination history of the participants against hepatitis immunization. On the other hand, the independent variables of the study were socio-demographic factors of the respondents (age, sex, marital status, working experience, etc.), behavioral and organizational factors (such as training of IPC, Presence of IPC committee, health and safety supervision, availability of infection prevention guidelines in their working departments, availability of PPE, etc.), knowledge, and attitude of IPCs.

### Data collection tools and techniques

Data collection was performed using a structured questionnaire that was pre-tested and adapted from existing published articles and contextualized based on the study setting (16, 19–21). The questionnaire was initially created in English, then translated into Amharic, and subsequently back-translated into English to ensure consistency. The questionnaire included questions on respondents’ socio-demographic characteristics, as well as behavioral and institutional factors, knowledge, attitudes, and infection prevention and control (IPC) measures. Data were collected through face-to-face interviews and observations, carried out by four environmental health professionals under the supervision of two public health experts.

### Data measurements

To assess the IPC of the healthcare providers, a set of 12 IPC-related questions were used with the option of either yes or no options. A score of 1 point was given to respondents who reported performing the specific IPC, while a score of 0 was given to those who did not perform it. Therefore, the overall score for IPC ranges from 0 to 12 and respondents who scored 80% or above were declared as having good infection prevention practices. On the contrary, respondents who scored below 80% were considered to have poor adherences infection prevention practices (22, 23).

Knowledge of IPC was measured using 10 questions with either “correct” or “incorrect” responses. For each knowledge-based question, respondents were given 1 point was given for correct response and 0 for an incorrect response. The overall knowledge score of the respondents were in the range of 0 to 10. Respondents who scored 80% or above were considered as having a good knowledge on adherence of infection prevention practices. On the contrary, HCWs who scored below 80% were considered as poor knowledge of IPC (22, 23).

Attitude HCWs on IPC was assessed using 10 items using a Likert scale with three options: disagree, agree, and neutral. For positive statements, those who responded “agree” were given three points, “neutral” responses were given two points, and “disagree” responses were given one point and vice versa for negative statements. Generally, respondents’ attitude scores ranged from 10 to 30 and those who scored 80% or higher were considered to have a favorable attitude whereas scored less than 80% were considered as having unfavorable attitude (23).

### Data quality assurance

Before starting the final data collection, a two-day training was given for both data collectors and supervisors. The contents of the training includes study objectives, clarity of the questionnaire, data collection procedures, and other related points. The content validity of the questionnaire was assessed using a pre-test with 5% of healthcare workers (21 HCWs) at Woldia General Hospital. Feedback from this pre-test was taken into account for final questionnaire, leading to necessary amendment of the questionnaire. During the process, close supervision was implemented to intervene any issues, with prompt corrections made for any incomplete or missing data to ensure data quality. Additionally, a random sample of 10% was selected for an extra data entry check to reduce potential errors.

### Data management and analysis

Data were entered into Epi-Data version 4.6 and exported to the Statistical Package for Social Sciences (SPSS) version 25 for data cleaning and analysis. The study findings were presented using descriptive statistics based on the nature of the data. The association between independent and dependent variables was assessed through binary logistic regression analysis, utilizing a 95% confidence interval. The strength of the association was expressed as odds ratios (OR) along with their corresponding 95% confidence intervals (CI). In bi-variable logistic regression analysis, independent variables with *p*-values less than 0.25 were retained for multivariable logistic regression analysis to account for potential confounders. The adjusted odds ratio (AOR) with 95% confidence intervals and a *p*-value under 0.05 were used to determine the significance of associations in the multivariable model. Model fitness was tested using the Hosmer and Lemeshow test; a p-value greater than 0.05 indicated a good fit. Reliability coefficients were assessed using Cronbach’s alpha, with values for knowledge, attitude, and practices related to infection prevention being 0.875, 0.756, and 0.821, respectively, all exceeding 0.7, which indicates acceptable reliability.

## Results

### Socio-demographic, behavioral, and organization-related characteristics of the HCWs

This study included 408 healthcare workers with a response rate of 96.7%. The mean age of the respondents was 33.03 ± 6 years. The majority, 50.5% (206), were female, and 45.8% (187) belonged to the age group of 31 to 40 years old. The majority of the respondents’ married 61.3% (250), and 43.4% (177) had an educational qualification of master’s degree and above. One-third 32.8% (134) of the HCWs have received training on infection prevention practices and 58.8% (240) had functional infection prevention committees while 74.5% (304) did not have infection prevention guidelines in their working healthcare facilities. Finally, more than two-thirds 68.9% (281) of the healthcare workers reported the lack of personal protective equipment in their working departments of healthcare facilities (Table 1).

**Table 1:** Socio-demographic, behavioral, and organizational characteristics of the healthcare workers in South Wollo Zone, Northeastern Ethiopia, following the Northern Ethiopia conflict from June 1 to 30, 2022 (*n* = 408).

Variable	Category	Frequency (*n*)	Percentage (%)
Sex	Male	202	49.5
	Female	206	50.5
Age	20–30	173	42.2
	31–40	187	45.8
	41 and above	48	11.8
Marital Status	Single	120	29.4
	Married	250	61.3
	Divorced	38	9.3
Religion	Muslim	181	44.4
	Orthodox	211	51.7
	Protestant	16	3.9
Working unit	Surgical	57	14.0
	Medical	78	19.1
	Pediatrics	55	13.5
	Gyn obese	58	14.2
	Laboratory	59	14.5
	Clinics	54	13.2
	Others	57	11.5
Educational status	Diploma	56	13.7
	BSc degree	175	42.9
	Master and above	177	43.4
Working experience	>5 years	241	59.1
	2–5 years	118	28.9
	<2 years	49	12.0
Professions	Doctor	109	26.7
	Midwife	46	11.3
	Nurse	102	31.4
	Laboratory	41	10.0
	Pharmacy	28	6.9
	Health officer	39	9.6
	Others	17	4.2
Do you have a history of chronic medical illness?	Yes	81	19.9
	No	327	80.1
Average monthly income	Less than or equal to 5,000	162	39.7
	5,001–7,000	111	27.2
	7,001 and above	135	33.1
Did you take IPC training after the occurrences of the Northern Ethiopia conflict?	Yes	134	32.8
	No	274	67.2
Is there a functional Infection prevention committee after the northern Ethiopia conflict?	Yes	168	41.2
	No	240	58.8
Do you have IPCs guidelines in your working unit/department?	Yes	104	25.5
	No	304	74.5
Do you have sufficient supply of PPEs in your working department/unit of the healthcare facility?	Yes	127	31.1
	No	281	68.9

### Knowledge of the HCWs on infection prevention practices

In this study, a substantial most of the participants, 96.1% (392) of participants had good knowledge of IPC. The average knowledge score of the respondents was 8.8 ± 1.0, with scores ranging from 7 to 10. Additionally, nearly one-third, 36.0% of the respondents knew that wearing gloves cannot replace the use of hand washing. It was found that only two-thirds of the participants, 273 (66.9%), recognized the need for administering post-exposure prophylaxis within 72 h to prevent the transmission of HCAIs. Moreover, less than half of the respondents, 165 (40.4%), were knowledgeable about the increased risk of nosocomial infections associated with an extended healthcare stay (Table 2).

**Table 2:** Healthcare worker’s knowledge of infection prevention practices in South Wollo Zone, Northeastern Ethiopia, following the northern Ethiopia conflict from June 1 to 30, 2022.

Knowledge item question	Correct	Incorrect
Disinfection prevents HCAIs.	403 (98.8)	5 (1.2)
Hand hygiene is the most effective method to prevent HCAIs.	404 (99.0)	4 (1.0)
Sterile gloves are the most effective tool to prevent HCAIs.	408 (100)	0 (0%)
Wearing gloves eliminates the use of hand washing.	261(64.0)	147 (36.0)
Prolonged hospital stay increases the risk of hospital-acquired infection.	165 (40.4)	243 (59.6)
Standard Precautions are taken to prevent Nosocomial infections.	278 (68.1)	130 (31.9)
All health workers and patients are considered potentially infectious.	379 (92.9)	29 (7.1)
Proper handling of working equipment reduces the risk of contamination.	363 (89.0)	45(11.0)
HAIs can be transmitted through blood and body fluid contamination.	276 (67.6)	132 (32.4)
Post-exposure prophylaxis should begin within 72 h.	273 (66.9)	135 (30.1)

### Attitude of HCWs on infection prevention practices

This study revealed that slightly more than three-quarters, 315 (77.2%) of the respondents showed a favorable attitude toward infection prevention practices. The mean score of attitude was 24.7 ± 1.6, out of a total score of 30. Approximately, one-third 127 (31.1%) of the respondents agreed that implementing standard operating procedures in healthcare settings reduces the risk of HCAIs. Moreover, slightly more than three-quarters of 79.9% (326) of the respondents agreed that proper ventilation through the opening of doors and windows can minimize the risk of HAIs. Finally, slightly more than one-third, 39.0% (159) of the participants acknowledged that nosocomial infections have serious consequences for healthcare providers (HCPs) (Table 3).

**Table 3:** Attitude of HCWs on infection prevention practices in South Wollo Zone, Northeastern Ethiopia, following the Northern Ethiopia conflict from June 1 to 30, 2022.

Attitude related questions	Agree	Neutral	Disagree
Do you agree that using the standard operating procedure reduces the risk of HCAIs?	127 (31.1)	203 (49.8)	78 (19.1)
Do you agree that ventilation of the ward by opening windows and doors can reduce the risk of infection transmission?	326 (79.9)	63 (15.4)	19 (4.7)
Do you agree that using of PPEs can reduce HCAIs?	396 (97.1)	12 (2.9)	0 (0)
Do you agree that hand washing before and after contact with patients is important?	233 (57.1)	136 (33.3)	39 (9.6)
Do you agree that healthcare facilities can be sources of HCAIs?	93 (22.8)	261(64.0)	54 (13.2)
Do you agree that recapping is the cause of needle prick injury?	250 (61.3)	143 (35.0)	15 (3.7)
Do you agree that using biohazard material is better for waste management?	382 (93.6)	26 (6.4)	0 (0)
Do you agree that patients’ awareness of the transmission of microorganisms may reduce the risk of HCAIs?	390 (95.6)	14 (3.4)	4 (1.0)
Do you agree that recapping is the cause of needle prick injury?	199 (48.8)	209 (51.2)	0 (0)
Do you agree that nosocomial infections may cause a serious outcome for HCWs?	159 (39.0)	220 (53.9)	29 (7.1)

### Infection prevention practice on HCWs

This study revealed that slightly more than half, 53.7% (219) of the respondents demonstrated safe infection prevention practices. The average score for infection prevention practices was 19.5 ± 1.4 (SD). Among all participants, 75.5% (308) reported washing their hands before providing patient care, 81.4% (334) used soap and water for hand washing, and 96.8% (395) washed their hands after coming into contact with body fluids. However, less than three-quarters of the participants, 72.8% (297), practiced segregation of healthcare waste at the point of generation based on its characteristics. Additionally, half of the respondents 49.5% (202), disposed of needles or sharps wastes in safety boxes and discarded these boxes when they were three-quarters full. Finally, less than two-thirds of the respondents, 60.3% (246), had received vaccination against the Hepatitis B virus (Table 4).

**Table 4:** Infection prevention practice among HCWs in South Wollo Zone, Northeastern Ethiopia, following the Northern Ethiopia conflict from June 1 to 30, 2022.

Infection prevention practice-related questions	Yes	No
Wash your hands always with water and soap after taking a sample	334 (81.9)	74 (18.1)
Wash your hands immediately when you come in to contact with blood, body fluid, and other contaminated items	395 (96.8)	13 (3.2)
Wash your hands after removing gloves	388 (95.1)	20 (4.9)
Wash your hands before and after contact with patients	308 (75.5)	100 (24.5)
Discard sharp needles in a safety box	202 (49.5)	206 (50.5)
Recap needles before disposal	191 (46.8)	217 (53.2)
Wear a gown properly for every procedure	277 (67.9)	131 (32.1)
Change gloves before starting handling new patient	300 (73.5)	108 (26.5)
segregate healthcare wastes at its point of generation based on their characteristics	297 (72.8)	111 (27.2)
Put on a mask and glasses when performing invasive and body fluid procedures	398 (97.5)	10 (2.5)
Provide health education to patients about HAIs	174 (42.6)	234 (57.4)
Take vaccines against Hepatitis B virus	246 (60.3)	162 (39.7)

### Factors associated with infection prevention practices among HCPs

In the multivariable logistic regression analysis, receiving training on IPC, the presence of a functional infection prevention committee, and the availability of infection prevention guidelines and personal protective equipment in the working departments of their healthcare settings were factors significantly associated with IPC of Healthcare workers.

HCWs who had received training on IPC had 2.662 times better IPCs compared to those who had not received the training (adjusted odds ratio [AOR] = 2.662, 95% confidence interval [CI]: 1.361, 5.120). Similarly, respondents with a functional infection prevention committee in their working departments had 2.203 times better IPCs compared to those who did not have such a committee (AOR = 2.203, 95% CI: 1.359, 3.572). Additionally, HCWs with infection prevention guidelines in their working departments had 2.090 times better infection prevention practices compared to those who did not have access to these guidelines (AOR = 2.090, 95% CI: 1.013, 4.312). Finally, respondents who had sufficient supplies of PPE in their working departments were 2.773 times more likely to implement safe IPC compared to those who did not have an adequate supply of these essential PPEs (AOR = 2.773, 95% CI: 1.560–4.929) (Table 5).

**Table 5:** Factors associated with infection prevention practice among healthcare workers in South Wollo Zone, Northeastern Ethiopia, following the Northern Ethiopia conflict, June 1–30, 2022.

Variable		Infection prevention practice		COR (95%CI)	AOR (95%CI)	*P*-value
		Good	Poor			
Age	20–30	97	76	Ref	Ref	
	31–40	84	103	0.639 (0.421–0.969)	0.663 (0.372–1.183)	0.164
	41 and above	38	10	2.977(1.394–6.357)	2.108(0.886–5.015)	0.092
Religion	Muslim	185	146	2.640 (1.283–5.433)	1.797 (0.772–4.183)	0.174
	Orthodox	22	18	2.546 (1.006–6.443)	1.079 (0.363–3.208)	0.892
	Protestant	12	25	Ref	Ref	
Working unit	Surgical	29	28	1.828 (0.830–4.026)	1.228 (0.485–3.104)	0.665
	Medical	49	29	2.982 (1.406–6.322)	1.384 (0.586–3.272)	0.459
	Paediatrics	34	21	2.857 (1.276–6.398)	1.728 (0.699–4.272)	0.236
	Gyn obese	30	28	1.891(0.861–4.153)	1.775 (0.705–4.473)	0.223
	Laboratory	50	9	9.804 (3.883–24.752)	1.728 (0.717–3.019)	0.321
	Clinics	10	44	0.401(0.162–0.995)	1.230 (0.820–2.646)	0.426
	Others	17	30	Ref	Ref	
Did you take IPC training?	Yes	99	35	3.630 (2.307–5.712)	2.662 (1.361–5.120)	0.004*
	No	120	154	Ref	Ref	
Is there functional IPC committee	Yes	106	62	1.921 (1.284–2.875)	2.203 (1.359–3.572)	0.001*
	No	113	127	Ref	Ref	
Presence of IPC guidelines	Yes	74	30	2.705(1.673–4.372)	2.090 (1.013–4.312)	0.046*
	No	145	159	Ref	Ref	
Presence sufficient PPEs	Yes	168	113	2.216 (1.445–3.397)	2,773 (1.560–4.929)	0.001*
	No	51	76	Ref	Ref	
Attitude	Negative	39	54	Ref	Ref	
	Positive	180	135	1.846 (1.156–2.949)	1.521(0.859–2.693)	0.150

Ref = reference variables ^*^ Factors significantly associated with infection prevention practice at 95% CI.

## Discussion

In Africa, the combination of endemic poverty and fragile health systems magnifies the adverse impacts of armed conflicts in the region. Conflict-prone areas are highly vulnerable to the re-emergence of infectious diseases caused by a shortage of healthcare personnel, inadequately equipped facilities, disrupted medical supply chains, limited access to health services, including preventive and curative interventions, and inadequate water and sanitation facilities (5, 24). The impact of these diseases has been further intensified by the presence of armed conflicts, resulting in severe and long-lasting consequences (5).

Globally, HCAIs continue to be a significant contributor to patient illness and death. Therefore, Implementation of a good IPC plays a crucial in ensuring the quality of care and safeguarding healthcare workers, patients, and communities from substantial risks (15, 25). The results of this study indicated that the respondents’ overall knowledge score regarding infection prevention practices was 88%, which is in line with studies done in Ethiopia (84.6%) (10). However, this score was higher compared to studies conducted in Ethiopia (76%) (26), (53.7%) (27), and Nigeria (16.6%) (28). More than three-quarters (86.1%) of the participating healthcare workers (HCWs) showed good knowledge, which aligns with studies conducted in Saudi Arabia (81%) (29), and Ethiopia (86% (30), 84.7% (10)). Conversely, it was lower compared to studies conducted in Bangladesh (37.5%) (31), Trinidad and Tobago (20.3%) (32), Palestine (53.9%) (22), Saudi Arabia (67.6%) (23), and Ethiopia (53.7% (27), 55.4% (33), and 60.4%) (34). However, it should be noted that this finding was higher than studies conducted in Ethiopia (90%) (11), (95.19%) (35). The variations observed in these findings may be attributed to differences in sample size, study context, composition of study participants, and the availability of ongoing awareness programs on infection prevention practices conducted by healthcare workers.

This finding also disclosed that approximately three-quarters of the participants showed a positive attitude towards infection prevention, which aligns with a study conducted in Ethiopia (76.4%) (30). However, this finding was slightly lower compared to studies conducted in Saudi Arabia (82%) (29) and Ethiopia (83.3%) (33). On the contrary, this finding was it was higher than studies conducted in Trinidad and Tobago (46.7%) (32), Saudi Arabia (61.5%) (23), and Ethiopia (57.2%) (11).These differences may be attributed due to variations in the study setting, duration, and the socio-demographic characteristics of the study populations.

More than half (53.7%) of the respondents reported safe infection prevention practices which was lower than studies done in Riyadh, Saudi Arabia (59%) (29), Palestinian (91.1%) (22), Saudi Arabia (61.5%) (23), and Ethiopia (60.4%) (34), (87.5%) (35), and (66.1%) (33). On the contrary, this result was higher than studies conducted in Bangladesh (39.1%) (31), Trinidad and Tobago (44.0%) (32), and Ethiopia (36.5%) (27), (36%) (11), (36.7%) (19). These variations could be attributed to variations in the composition of the study participants, the level of healthcare settings (being health centers, hospitals, and others) the location and timing of the study, the availability of essential PPEs, and the method of data collection.

Lack of resources in healthcare settings emerged as a significant challenge to implement safe IPCs, particularly in conflict-affected settings due to the destruction of essential healthcare facilities. These facilities include a lack of hospital furniture, limited availability of medicines, essential supplies for IPCs, including (PPEs), hand hygiene products, and cleaning equipment. Therefore, the lack of PPEs in healthcare settings plays a crucial role in the spread of healthcare-associated infections (9, 36).

The current finding revealed that lack of PPEs was one of the factors in implementing a safe IPC among healthcare workers which was supported by previous studies conducted in Ethiopia (10, 19, 32), Bangladesh (31), and India (36). Despite the responses to the ongoing coronavirus pandemic providing important lessors for IPC programs in resource-limited and conflict-affected settings, implementing safe infection prevention practices is still in a backward position due to the lack of essential facilities and services due to the ongoing destruction in the conflict process (9). Therefore, both parties of the combatants should ensure the safety of these essential facilities to provide better healthcare services for both parties of the conflict and the communities as a whole. Therefore, concerned governmental and non-governmental organizations should closely follow the process and facilitate and design a timely intervention (9).

The provision of continuous awareness creation sessions on infection prevention practices among healthcare providers plays a vital role in implementing safe infection prevention practices to reduce the burden of HCAIs (36). In conflict-affected regions, regular in-service training, including hands-on practical sessions and consistent monitoring and supervision of staff, is acknowledged as an effective strategy to enhance IPC practices (9). The finding of this study also supports that the implementation of updated training on infection prevention practices was another factor which affected in implementation of safe infection prevention practices which was supported by other previous findings done in Ethiopia (10, 26, 27), India (36), and Saudi Arabia (23). Training programs based on updated guidelines have the potential to enhance the knowledge, skills, and confidence of healthcare workers, enabling them to effectively implement better infection prevention practices, particularly in resource-limited settings due to the damage of essential facilities (9).

WHO released guidelines regarding IPC at national and facility levels which are designed to be applicable in all countries and healthcare settings. Nevertheless, its implementation universally can significantly vary based on the unique circumstances and conditions of each situation (9). The results of this study demonstrate that the utilization of infection prevention guidelines in working departments served as an additional contributing factor in the successful implementation of effective infection prevention practices. This finding aligns with previous research conducted in Ethiopia, (10, 27, 33), Riyadh, Saudi Arabia (29), and India (36) highlighting the importance of following established guidelines to ensure the safety of infection prevention measures. However, during periods of critical circumstances such as armed conflicts, the implementation of these guidelines becomes challenging due to the destruction of vital facilities. In such situations, the presence of a functional IPC committee becomes crucial as it offers a platform for interdisciplinary involvement, cooperation, and the exchange of information. These facilitates are fundamental for the successful implementation of safe infection prevention practices despite the difficult circumstances (22, 34). The availability of an infection prevention and control (IPC) committee is a fundamental initial action in establishing an IPC program, especially in settings impacted by conflict (9). The results of this study indicate that the presence of a functional IPC committee is a significant influencing factor in ensuring the safe implementation of infection prevention measures. This finding is consistent with previous research conducted in various conflict-affected countries, further supporting the importance of having a well-functioning IPC committee in such contexts (9). The findings of this study will provide insight into humanitarian interventions and serve as input for government and other stakeholders in designing evidence-based health interventions for conflict-affected areas. It will also serve as baseline evidence for further investigation in the area.

### Limitations of the study

This study has certain limitations. Initially, the use of a cross-sectional design makes it challenging to establish a definitive cause-and-effect relationship between the dependent and independent variables under investigation. Secondly, the measurement of infection prevention practices relied on self-reported responses from the participants, which may introduce the possibility of social desirability bias influencing the accuracy of the data.

## Conclusion

In conclusion, slightly more than half of the healthcare workers exhibited good adherence to infection prevention practices. Regarding to factors, existence of a functioning infection prevention committee, utilizing of infection prevention guidelines in their working department, availability of personal protective equipment (PPE) in their departments, and received training on infection prevention and control were factors significantly associated with adherences of infection prevention among healthcare workers. Therefore, it is recommended that ongoing awareness program should be implemented to enhance their knowledge and practices. Additionally, donor organizations in collaboration with government organization should ensure a sufficient supply of PPE to support healthcare workers in improving their infection prevention efforts during this critical period. For a more comprehensive understanding, future research should adopt a mixed-methods approach that integrates both qualitative and quantitative methods to gather in-depth information.

## Data Availability

The raw data supporting the conclusions of this article will be made available by the authors, without undue reservation.

## References

[1] Arage MW Kumsa H Asfaw MS Kassaw AT Dagnaw EM Tunta A. Exploring the health consequences of armed conflict: the perspective of Northeast Ethiopia, 2022: a qualitative study. BMC Public Health. (2023) 23:2078. doi: 10.1186/s12889-023-16983-z, PMID: 37875885 PMC10594710

[2] Arage MW Kumsa H Asfaw MS Kassaw AT Dagnaw EM Tunta A. Assessing the health consequences of northern Ethiopian armed confict, 2022. J Public Health Policy. (2024) 45:43–57. doi: 10.1057/s41271-023-00464-z, PMID: 38310169 PMC10920422

[3] Ekzayez A Ahmad YA Alhaleb H Checchi F. The impact of armed confict on utilisation of health services in north-West Syria: an observational study. Confl Heal. (2021) 15:91. doi: 10.1186/s13031-021-00429-7, PMID: 34906188 PMC8670035

[4] Awojobi ON. The impact of conflict on health outcomes: a systematic evidence from sub-Saharan Africa. Mgbakoigba J Afr Stud. (2019) 8:88–100.

[5] Tekulu F.B. Gebre H.T. Hailu H.G. Asgedom D.B. Desta G.M. Shifare T.H.et.al. The effect of war on health institutions of eastern Tigray zone, Tigray state, Ethiopia. Ethiopia J Health Commun. (2023). [Preprint].

[6] Bardossy AC Zervos J Zervos M. Preventing Hospitalacquired infections in low-income and middle-income countries: impact, gaps, and opportunities. Infect Dis Clin N Am. (2016) 30:805–18. doi: 10.1016/j.idc.2016.04.006 27515149

[7] Kodo TK Kidie AB Merecho TH Tiruneh MG Yayeh BM Getaneh BA. The impact of armed conflict on services and outcomes related to maternal and reproductive health in north Wollo, Amhara, Ethiopia: a qualitative study. Int J Women's Health. (2024) 16:1055. doi: 10.2147/IJWH.S457529 38863520 PMC11166144

[8] World Health Organization. Global strategy on infection prevention and control. Geneva: World Health Organization (2023).

[9] Lowe H Woodd S Lange IS Janjanin S Barnet J Graham W. Challenges and opportunities for infection prevention and control in hospitals in confict-afected settings: a qualitative study. Confict Health. (2021) 15:94. doi: 10.1186/s13031-021-00428-8, PMID: 34930364 PMC8686079

[10] Desta M Ayenew T Sitotaw N Tegegne N Dires M Getie M. Knowledge, practice and associated factors of infection prevention among healthcare workers in Debre Markos referral hospital, Northwest Ethiopia. BMC Health Serv Res. (2018) 18:465. doi: 10.1186/s12913-018-3277-5, PMID: 29914477 PMC6006704

[11] Bayleyegn B Mehari A Damtie D Negash M. Knowledge, attitude and practice on hospital-acquired infection prevention and associated factors among healthcare Workers at University of Gondar comprehensive specialized hospital, Northwest Ethiopia. Infect Drug Resist. (2021) 14:259. doi: 10.2147/IDR.S290992 33536767 PMC7850400

[12] Tirivanhu C. Estele M. Elizabeth T. Chipo C.. Knowledge and practices of healthcare workers in prevention and control of hospital-acquired infections in the maternity Department at Bindura Provincial Hospital, (Zimbabwe: International Journal of Infection Control). (2023).

[13] Abalkhail A Alslamah T. Institutional factors associated with infection prevention and control practices globally during the infectious pandemics in resource-limited settings. Vaccine. (1811) 10:10. doi: 10.3390/vaccines10111811, PMID: 36366320 PMC9696365

[14] Alemu AY Endalew A Bayih WA. The burden of healthcare-associated infection in Ethiopia: a systematic review and meta-analysis. Tropical. Med Health. (2020) 48:77. doi: 10.1186/s41182-020-00263-2, PMID: 32939151 PMC7487565

[15] Tesfaye AH Mekonnen TH Desye B Yenealem DG. Infection prevention and control practices and associated factors among healthcare cleaners in Gondar City: an analysis of a cross-sectional survey in Ethiopia. Risk Manag Healthc Policy. (2023) 16:1317–30. doi: 10.2147/RMHP.S419110 37492624 PMC10363670

[16] Murray CJL King G Lopez AD TomijimaN KEG. Armed conflict as a public health problem education and debate. BMJ. (2002) 324:346–9. doi: 10.1136/bmj.324.7333.346 11834565 PMC1122272

[17] Hassen SL Astatkie A Mekonnen TC Bogale GG. Survival status and its determinants among under-five children with severe acute malnutrition admitted to inpatient therapeutic feeding centers in south Wollo zone, Amhara region, Ethiopia. J Nutr Metab. (2019) 2019:1–9. doi: 10.1155/2019/2643531, PMID: 31049224 PMC6462333

[18] Abebe A Federal Democratic Republic of Ethiopia central statistical agency population projection of Ethiopia for all regions at Wereda level from 2014–2017. Federal Demographic Republic of Ethiopia Central Statistical Agency: Population Projection of Ethiopia for All Regions At Wereda Level from 2014 – 2017. (2013).

[19] Zenbaba D Sahiledengle B Bogale D. Practices of healthcare workers regarding infection prevention in bale zone hospitals, Southeast Ethiopia. Hindawi Adv Public Health. 2020:4198081. doi: 10.1155/2020/4198081

[20] Jiménez A Livsey J Åhlén I Scharp C Takane M. Global assessment of accountability in water and sanitation services using GLAAS data. Water Alternat. (2018) 11:238–59.

[21] Assefa J Diress G Adane S. Infection prevention knowledge, practice, and its associated factors among healthcare providers in primary healthcare unit of Wogdie District, Northeast Ethiopia, 2019: a cross-sectional study. Antimicrob Resist Infect Control. (2020) 9:136. doi: 10.1186/s13756-020-00802-w, PMID: 32807230 PMC7433044

[22] Fashafsheh I Ayed A Eqtait F Harazneh L. Knowledge and practice of nursing staff towards infection control measures in the Palestinian hospitals. J Educ Pract. (2015) 6:79–90.

[23] Abalkhail A Al Imam MH Elmosaad YM Jaber MF Hosis KA Alhumaydhi FA. Knowledge, attitude, and practice of standard infection control precautions among health-Care Workers in a University Hospital in Qassim, Saudi Arabia: a cross-sectional survey. Int J Environ Res Public Health. (2021) 18:11831. doi: 10.3390/ijerph182211831, PMID: 34831585 PMC8624606

[24] Haddison EC Chia E Julius CE Kagina BM. Health services utilisation before and during an armed conflict; experiences from the southwest region of Cameroon. Open Public Health J. (2020) 13:547–54. doi: 10.2174/1874944502013010547

[25] Sifir CK. Infection prevention practices and associated factors among healthcare Workers in Governmental Hospitals in Addis Ababa, Ethiopia. Adv Sex Reprod Health Res. (2022) 2:190–6.

[26] Amsalu A Kassaye H. Healthcare workers’ compliance and factors for infection prevention and control precautions at Debre Tabor referral hospital, Ethiopia. PAMJ One Health. [research square]. (2022) 7:32202. doi: 10.11604/pamj-oh.2022.7.35.32202

[27] Geberemariyam BS Donka GM Wordofa B. Assessment of knowledge and practices of healthcare workers towards infection prevention and associated factors in healthcare facilities of west Arsi District, Southeast Ethiopia: a facility-based cross-sectional study. Arch Public Health. 76:69. doi: 10.1186/s13690-018-0314-0 PMC6231270 30455882

[28] Oluwagbemiga AO Akinsete SJ Ana GR Gunseye OO. Knowledge, attitude and self-reported practice of healthcare workers on infection control in a health facility in Akure, Nigeria. Int J Infect Control. (2021) 17:20818. doi: 10.3396/ijic.v17.20818

[29] Alshathri N. Knowledge, attitude and practice regarding infection control measures among healthcare Workers at King Khaled eye Specialist Hospital (KKESH) in Riyadh, KSA. Res Squ. (2021). doi: 10.21203/rs.3.rs-958840/v1

[30] Gezie H Leta E Admasu F Gedamu S Dires A. Health care workers' knowledge, attitude, and practice towards hospital-acquired infection prevention at Dessie referral hospital, Northeast Ethiopia. Clin J Nurs Care Pract. (2019) 3:059–63. doi: 10.29328/journal.cjncp.1001019

[31] Hsan K Islam MS Islam MZ Awal N Gozal D MMM K. Healthcare providers infection prevention practices and associated factors in community clinics in Bangladesh: a cross-sectional study. PLoS Glob Public Health. (2022) 2:e0000574. doi: 10.1371/journal.pgph.0000574, PMID: 36962382 PMC10022338

[32] Unakal CG Nathaniel A Keagan B Alexandria B Lauralee B Chatoo VC. Assessment of knowledge, attitudes, and practices towards infection prevention among healthcare workers in Trinidad and Tobago. International journal of community medicine and public health Unakal CG et al. Int J Community Med Public Health. (2017) 4:2240–7. doi: 10.18203/2394-6040.ijcmph20172813

[33] Sahiledengle B Gebresilassie A Hiko D Getahun T. Infection prevention practices and associated factors among healthcare Workers in Governmental Healthcare Facilities in Addis Ababa, Ethiopia. Ethiop J Sci. (2018) 28:177–86. doi: 10.4314/ejhs.v28i2.9, PMID: 29983515 PMC6016341

[34] Sado MH Fasil N Bekere A. Assessment of infection prevention practice and associated factors among healthcare providers in the case of Bishoftu referral hospital. Clin Med Res. (2021) 10:212–24. doi: 10.11648/j.cmr.20211006.17

[35] Alemayehu R Ahmed K Sada O. Assessment of knowledge and practice on infection prevention among health Care Workers at Dessie Referral Hospital, Amhara region, south Wollo zone, North East Ethiopia. J Community Med Health Educ. (2016) 6:487. doi: 10.4172/2161-0711.1000487

[36] Thazha SK Cruz JP Alquwez N Scaria B Rengan SS Almazan JU. Infection prevention and control awareness, attitudes, and practices among healthcare professionals in South India. J Infect Dev Ctries. (2022) 16:659–67. doi: 10.3855/jidc.14746, PMID: 35544628

